# The Karnofsky Performance Status at Discharge Is a Prognostic Indicator of Life Expectancy in Patients With Glioblastoma

**DOI:** 10.7759/cureus.66226

**Published:** 2024-08-05

**Authors:** Shogo Sasaki, Shinji Tsukamoto, Yukako Ishida, Yasuyo Kobayashi, Yusuke Inagaki, Tomoo Mano, Tetsuro Kitamura, Naoto Seriu, Ichiro Nakagawa, Akira Kido

**Affiliations:** 1 Rehabilitation Medicine, Nara Medical University, Kashihara, JPN; 2 Orthopedic Surgery, Nara Medical University, Kashihara, JPN; 3 Neurosurgery, Nara Medical University, Kashihara, JPN

**Keywords:** life expectancy, prognosis, the structure of disability, glioblastoma, karnofsky performance score

## Abstract

Background

Glioblastoma (GBM) is the most frequent invasive brain tumor and a rapidly progressive disease with a poor prognosis that predominantly affects middle-aged and older adults. The relationship between daily functioning and prognosis in patients with GBM will become more important as advances in multimodality treatment are expected to increase the number of long-term survivors.

Methods

Sixty-seven patients were initially diagnosed with GBM at our hospital between December 2013 and December 2022. All patients were divided into two groups: those who survived for one year or longer from the date of discharge (Group A) and those who died within one year from the date of discharge (Group B). Muscle strength, nutritional status, and Karnofsky Performance Status (KPS) were examined upon admission (p1), post-surgery (p2), and discharge (p3), and their relationships with prognosis were investigated.

Results

Group A was significantly younger than Group B, with a significant difference in the total radiation dose. There were no significant differences in the anatomical tumor location, whether the tumor occurred on the left or right side, or tumor size. KPS at discharge (p3) and the degree of improvement in the KPS between p1 and p3 were associated with a good prognosis.

Conclusions

The KPS varies throughout the treatment. When considering the KPS as a prognostic indicator, the KPS at discharge is the most important, given the structure of the disability and the course of treatment for GBM.

## Introduction

Glioblastomas (GBMs) arise from both neural and glial progenitor cells [1]. GBM is also referred to as GBM multiforme due to its diverse morphology [2,3]. GBM is the most frequently observed invasive brain tumor, with a peak incidence in the 60s to 70s age group [4]. The mean overall survival (OS) is less than 18 months [5], and rapid progression is a clinical hallmark of this disease.

The standard of care for patients aged <70 years is maximally safe resection followed by radiation therapy with temozolomide (Stupp regimen) [6]. The Stupp regimen has been reported to prolong OS in older patients with good performance [7].

Prognostic indicators for patients include age at surgery, tumor size, site of tumor origin, nature of the tumor (multifocal or bilateral), and extent of resection (EOR) [8,9]. Molecular diagnoses are also critical. In particular, the presence or absence of methylation in the promoter regions of O^6^-methylguanine-DNA methyltransferase (MGMT) [10] and isocitrate dehydrogenase (IDH) is important [11]. MGMT is an enzyme that repairs alkylated DNA. Therefore, methylation of the MGMT promoter region assists in killing tumor cells using alkylating agents [12]. IDH, a rate-limiting enzyme in the citric acid cycle, functions in oxidative decarboxylation [13]. IDH mutation positivity has been considered a favorable prognostic factor in GBM. However, with the revision of the WHO brain tumor classification in 2021, what was previously classified as IDH-mutated GBM was removed from the GBM category [14]. As a result of this revision, all pathologically diagnosed GBMs are wild-type IDH.

While GBM generally has a poor prognosis, there are reports of long-term survivors exceeding three to five years, although the percentage is low [15]. The disability profile of GBM survivors has received increasing attention in recent years. The relationship between daily functioning and prognosis in patients with GBM will become even more important as advances in multidisciplinary treatment are expected to increase the number of long-term survivors. In addition to the clinical factors and molecular diagnoses already widely used as prognostic factors for patients with GBM, we hypothesized that the daily functioning score at discharge from the hospital might be a potential prognostic indicator for GBM based on our clinical experience. In this case, it can be combined with genetic testing, which has already been reported to provide a more precise indicator of the patient’s medical care.

Several reports have been published on changes in the Karnofsky Performance Status (KPS) [16] in patients with GBM and its relationship with prognosis [17,18]. The KPS changes in various ways during treatment, and therefore, when considering the KPS as a prognostic indicator, it must be determined at what point in the treatment process the change occurred. Considering the structure of disability in patients with GBM and the standard treatment process, we hypothesized that the KPS at the time of discharge from the hospital, especially after the completion of radical resection and radiation therapy, would be an appropriate prognostic indicator.

The purpose of this study is to determine at what stage of treatment the KPS score measured is a prognostic indicator for GBM patients.

## Materials and methods

Patients diagnosed with GBM at Nara Medical University Hospital in Kashihara, Japan, between December 2013 and December 2022, were included in this study. The age range of the patients was 32-92 years. The exclusion criteria were as follows: patients whose pathological findings did not lead to a definitive diagnosis; patients who did not receive standard treatment [6]; and patients who did not receive rehabilitation treatment from a therapist during hospitalization.

Patient background (sex, age, treatment, and number of days from discharge to date of death) and tumor characteristics (anatomical site of origin, whether it occurred on the left or right side, and tumor size) were obtained from medical records, and for patients who were alive as of May 2023, the observation period ranged from discharge to the present day.

Clinical findings included manual muscle testing (MMT) values for the upper and lower extremities [19], serum albumin levels (Alb), and the KPS. MMT, Alb, and KPS scores were examined at three time points: on admission (p1), post-surgery (p2), and at discharge (p3). MMT is a muscle assessment method that manually evaluates muscle weakness in individual muscles and is graded on a six-point scale from 0 to 5. Grade 0 is no contraction detected; grade 1 is a visible or palpable contraction detected without joint movement; grade 2 is the ability to move through the full range of motion only against gravity; grade 3 is defined as being able to move through the full range of motion against gravity; grade 4 is able to move through the full range of motion and against moderate resistance; and grade 5 is able to move through the full range of motion and against maximal resistance [19]. Upper extremity MMT was defined as the lowest MMT value of the bilateral shoulder, elbow, and wrist. Similarly, lower extremity MMT was defined as the lowest MMT value of the bilateral hip, knee, and ankle joints. KPS was measured by the patient’s ability to carry out normal activities or the degree of dependence on help and care. It is expressed as a percentage, and the criteria used are those given in Table 1 [16].

**Table 1:** KPS Source: Karnofsky et al. (1948) [16] KPS, Karnofsky Performance Status

%	Criteria
100	Normal; no complaints; no evidence of disease
90	Able to carry on normal activity; minor signs or symptoms of disease
80	Normal activity with effort; some signs or symptoms of disease
70	Cares for self; unable to carry on normal activity or to do active work
60	Requires occasional assistance but is able to care for most of his needs
50	Requires considerable assistance and frequent medical care
40	Disabled; requires special care and assistance
30	Severely disabled; hospitalization is indicated, although death is not imminent
20	Very sick; hospitalization necessary; active supportive treatment necessary
10	Moribund; fatal processes progressing rapidly
0	Dead

Improvement from admission to discharge (p3-p1) and post-surgery to discharge (p3-p2) was also investigated using MMT and KPS. All patients were divided into two groups: those who survived for one year or longer from the discharge date (Group A) and those who died within one year (Group B).

This study was approved by the Ethics Committee of Nara Medical University Hospital, and information was collected from medical records in accordance with the Declaration of Helsinki while maintaining patient anonymity and confidentiality.

Statistical analysis was performed using the Shapiro-Wilk test for normality, and the t-test and chi-square test with no correspondence between the two groups were used. A statistical significance level of p < 0.05 was considered significant. The statistical software used was IBM SPSS Statistics for Windows, Version 21.0 (Released 2012; IBM Corp., Armonk, NY, USA).

## Results

Between 2013 and December 2022, 67 patients were diagnosed with GBM at our hospital. We excluded ten patients who did not receive the standard treatment and two who did not receive rehabilitation therapy by a therapist during hospitalization. Therefore, 55 patients were included in the study. Of these, 43 patients survived more than one year after discharge (Group A), and 12 patients died within one year after discharge (Group B). The cause of death in this population was related to GBM in all patients.

Table 2 shows a comparison of patient backgrounds. In comparison, patients in Group A were significantly younger than those in Group B. There was a significant difference in the total radiation dose between the treatment modalities.

**Table 2:** Comparison of patients’ backgrounds between survival and deceased group Values are mean ± SD.

Parameter	Overall (n = 55)	Group A (n = 43)	Group B (n = 12)	p-Value
Age (years)	65.06 ± 14.09	62.33 ± 14.27	73.08 ± 9.43	p = 0.02*
Men	25	20	5	p = 0.77
Women (n)	30	23	7	
Lengths of hospital stay (days)	77.24 ± 27.41	78.91 ± 29.55	72.58 ± 16.88	p = 0.49
Survival period (days)	697.15 ± 529.56	821.00 ± 536.04	253.33 ± 61.14	p = 0.001*
Total radiation therapy (n)				p = 0.001*
60 Gy/30 fr	41	36	5	
40.05 Gy/15 fr	13	7	6	
25 Gy/5 fr	1	0	1	

Table 3 shows a comparison of the tumor characteristics. The two groups showed no significant differences in the tumor size or the anatomic site of tumor origin, regardless of whether it occurred on the left or right side.

**Table 3:** Comparison of tumor characteristics between survival and deceased group Values are mean ± SD.

Parameter	Overall (n = 55)	Group A (n = 43)	Group B (n = 12）	p-Value
Tumor size (mm)	40.06 ± 12.91	38.98 ± 12.51	43.00 ± 13.80	p = 0.35
Tumor location (n)				p = 0.47
Frontal	14	11	3	
Parietal	10	9	1	
Temporal	21	16	5	
Occipital	4	3	1	
Others	6	4	2	
Tumor hemisphere (n)				p = 0.55
Right	24	17	7	
Left	30	26	4	
Bilateral	1	0	1	

Table 4 shows a comparison of the clinical findings. There was no significant difference in MMT, but the Alb values at discharge were significantly higher in Group A than in Group B. A comparison of the KPS scores between the two groups yielded interesting results. This study measured the KPS at three time points: on admission (p1), post-surgery (p2), and at discharge (p3). Only the KPS score at discharge (p3) was associated with a good prognosis (Table 4). In addition, the degree of improvement in KPS, that is, the difference between KPS at discharge (p3) and KPS at admission (p1), was also associated with the prognosis.

**Table 4:** Comparison of clinical findings between survival and deceased group Alb, albumin; KPS, Karnofsky Performance Status; MMT, manual muscle testing

Parameter	Overall (n = 55)	Group A (n = 43)	Group B (n = 12)	p-Value
MMT				
Upper limb (median)				
Admission	3.93	3.98	3.83	p = 0.67
Post-surgery	3.63	3.77	3.17	p = 0.12
Discharge	3.89	4	3.58	p = 0.18
Discharge-admission	-0.04	0.02	-0.25	p = 0.25
Discharge-post-surgery	0.26	0.23	0.42	p = 0.43
Lower limb (median)				
Admission	3.91	3.95	3.83	p = 0.73
Post-surgery	3.57	3.7	3.25	p = 0.27
Discharge	3.87	4	3.5	p = 0.12
Discharge-admission	-0.04	0.05	-0.33	p = 0.14
Discharge-post-surgery	0.3	0.3	0.25	p = 0.81
Alb (median)				
Admission	4.23	4.24	4.15	p = 0.49
Post-surgery	3.35	3.39	3.32	p = 0.57
Discharge	3.8	3.83	3.49	p = 0.02*
KPS (median)				
Admission	57.04	57.91	55	p = 0.64
Post-surgery	39.44	40.7	36.67	p = 0.39
Discharge	59.63	62.79	49.17	p = 0.02*
Discharge-admission	2.59	4.88	-5.83	p = 0.05*
Discharge-post-surgery	20.19	22.09	12.5	p = 0.07

## Discussion

This study focused on the KPS, muscle strength, and nutritional status of patients with GBM at three time points (admission, post-surgery, and discharge) and investigated their relationship with prognosis. Patients with GBM receive treatment (on the Stupp regimen) for several months, from hospitalization to discharge, and their physical function and activity levels change significantly according to the course of treatment. Here, we present the structure of the disability and the treatment of GBM.

**Figure 1:**
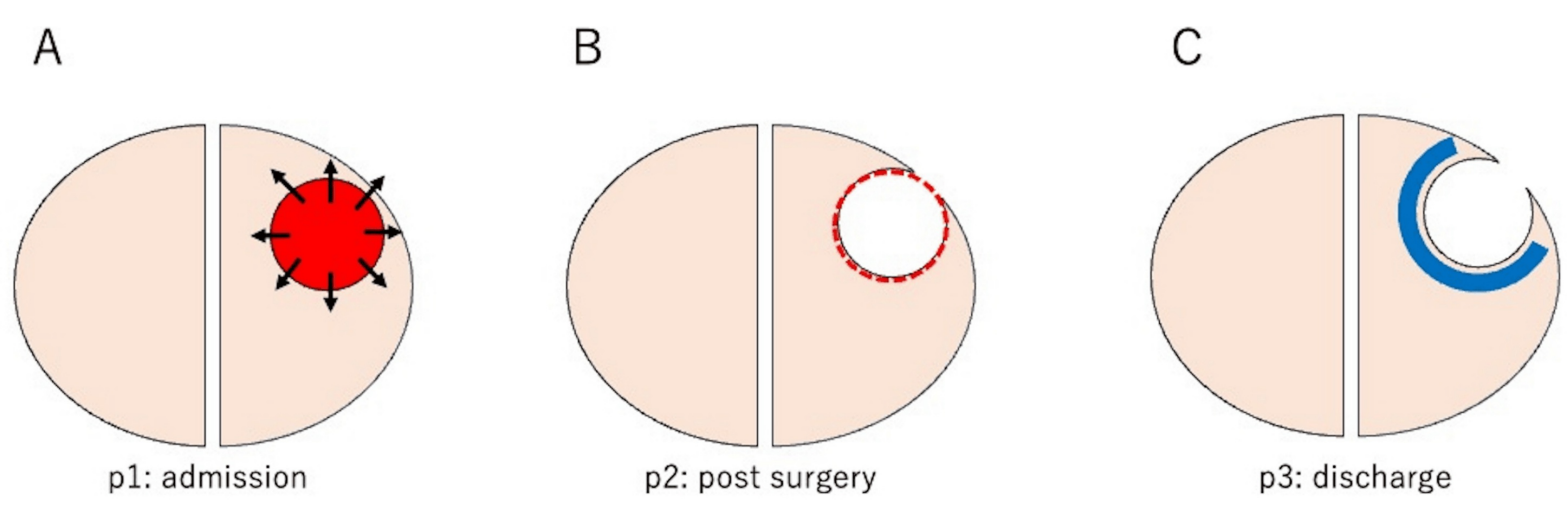
GBM treatment process The GBM treatment process is divided into three periods, where p1 represents the patient’s condition immediately after admission. Intracranial pressure was elevated due to the tumor volume (A). p2 represents the postsurgical resection state. (B) Inflammation and brain swelling due to surgical invasion. p3 represents the condition at the time of discharge. The blue area indicates residual tumors (C). This figure is the authors’ own creation. GBM, glioblastoma

The treatment of GBM is divided into three periods (p1-p3) [6], and we believe that the KPS score for patients with GBM changes significantly depending on the treatment period; p1 in Figure 1A represents the patient’s condition immediately after admission. In addition to focal cerebral symptoms due to tumor invasion, intracranial pressure is elevated due to tumor volume; p2 in Figure 1B represents the postsurgical resection state. Immediately after surgical resection, the patient often requires daily living assistance because of headaches and nausea caused by inflammation and brain swelling due to surgical intervention. Disabilities in this state are predominantly deficit symptoms due to resection. Including the tumor and eloquent areas is critical in defining deficit symptoms. The blue area in Figure 1C indicates the residual tumor. The fewer residual tumors, the better the functional and life prognosis. Therefore, sensitivity to drugs and radiation therapy determines the magnitude of the disability in this state.

**Figure 2:**
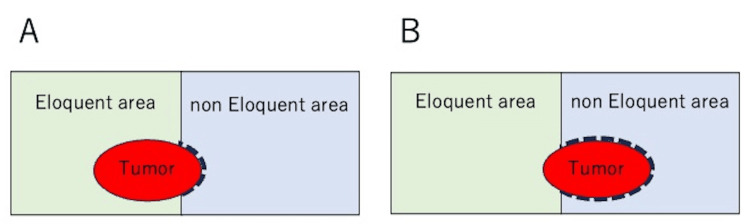
Relationship between tumor localization and inclusion of eloquent/non-eloquent areas The area where the radical margin could be secured was smaller when the eloquent area of the tumor was larger (A) than when the non-eloquent area of the tumor was larger (B). The radical margins are shown schematically with thick dotted lines. This figure is the authors’ own creation.

Figure 2 schematically illustrates the relationship between tumor localization and the inclusion of eloquent and non-eloquent areas. This inclusion is important for defining the deficiency symptoms when planning a maximally safe resection. This is because the area in which the radical margin could be secured was smaller when the eloquent area of the tumor was larger (Figure 2A) than when the non-eloquent area of the tumor was larger (Figure 2B). In most cases, intraoperative monitors are used to identify the eloquent area. However, postoperative neurological symptoms may unexpectedly appear. Surgical resection often results in the disappearance of symptoms other than focal as the increased intracranial pressure caused by the tumor, inflammation, and brain swelling caused by surgical intervention are resolved. During hospitalization, many patients achieve maximum residual function through rehabilitation therapy.

The results of this study show that the KPS at discharge (p3) and the degree of improvement in the KPS between p1 and p3 were associated with prognosis. Some previous reports have shown an association between admission and post-surgery KPSs and prognosis [20-23], while others have found an association between the KPS and prognosis but did not specify the point at which the KPS was measured [24].

Among these studies, Kawauchi et al. [21] identified both preoperative and postoperative KPS ≤60 as significant predictors of shorter survival. Conversely, Șerban et al. [22] and Liu et al. [23] emphasized the prognostic value of postoperative KPS, with Liu et al. [23] reporting that a postoperative KPS ≥80, along with total resection and adherence to the Stupp protocol, was a useful prognostic factor. Awad et al.’s report [20] aligns with our failure model, demonstrating that while the EOR alone is not a crucial predictor, aggressive surgical treatment to minimize postoperative residuals, coupled with maintaining a high postoperative KPS, may enhance patient survival.

Our clinical experience has encountered many patients whose KPSs change from admission to discharge. Therefore, considering the structure of disability and the treatment process described above, the degree of improvement from p1 to p3 or p3 after the completion of standard treatment should be regarded as essential when using the KPS as a prognostic indicator in GBM.

Limitations

This study has several limitations. First, it is a retrospective analysis conducted at a single institution. Second, the sample size is relatively small, with data spanning a decade (2013-2021) and care provided by multiple attending physicians for the 67 patients. A multicenter study is needed for a broader investigation. Third, there is an age difference between the groups, and age is a well-established prognostic factor in glioblastoma [5,10]. Prognosis may also be influenced by other factors, such as the tumor’s localization in eloquent versus non-eloquent areas.

## Conclusions

We found that the KPS at discharge serves as a valuable indicator of several prognostic factors. The correlation between prognosis and KPS at discharge (p3) observed in this study may partially reflect an age-related prognostic trajectory. Patients with GBM undergo treatment for several months, and their KPS can change over this period. The most significant prognostic factor is the improvement in the KPS score from admission to discharge and after completing treatment. Even patients with a low KPS score at admission might experience prolonged survival if their KPS score improves by discharge.
